# Raman Multi-Omic Snapshot and Statistical Validation of Structural Differences between Herpes Simplex Type I and Epstein–Barr Viruses

**DOI:** 10.3390/ijms242115567

**Published:** 2023-10-25

**Authors:** Giuseppe Pezzotti, Eriko Ohgitani, Hayata Imamura, Saki Ikegami, Masaharu Shin-Ya, Tetsuya Adachi, Keiji Adachi, Toshiro Yamamoto, Narisato Kanamura, Elia Marin, Wenliang Zhu, Koichiro Higasa, Yoshiki Yasukochi, Kazu Okuma, Osam Mazda

**Affiliations:** 1 Ceramic Physics Laboratory, Kyoto Institute of Technology, Sakyo-Ku, Matsugasaki, Kyoto 606-8585, Japan; hyt8888@outlook.jp (H.I.); b0151006@edu.kit.ac.jp (S.I.); wlzhu@kit.ac.jp (W.Z.); 2 Department of Molecular Genetics, Institute of Biomedical Science, Kansai Medical University, 2-5-1 Shin-Machi, Hirakata 573-1010, Japan; 3 Department of Immunology, Graduate School of Medical Science, Kyoto Prefectural University of Medicine, Kamigyo-Ku, 465 Kajii-Cho, Kyoto 602-8566, Japan; ohgitani@koto.kpu-m.ac.jp (E.O.); masaharu@koto.kpu-m.ac.jp (M.S.-Y.); t-adachi@koto.kpu-m.ac.jp (T.A.); mazda@koto.kpu-m.ac.jp (O.M.); 4 Department of Dental Medicine, Graduate School of Medical Science, Kyoto Prefectural University of Medicine, Kamigyo-Ku, Kyoto 602-8566, Japan; keiji922@koto.kpu-m.ac.jp (K.A.); yamamoto@koto.kpu-m.ac.jp (T.Y.); kanamura@koto.kpu-m.ac.jp (N.K.); 5 Department of Orthopedic Surgery, Tokyo Medical University, 6-7-1 Nishi-Shinjuku, Shinjuku-Ku, Tokyo 160-0023, Japan; 6 Department of Applied Science and Technology, Politecnico di Torino, Corso Duca degli Abruzzi 24, 10129 Torino, Italy; 7 Department of Molecular Science and Nanosystems, Ca’ Foscari University of Venice, Via Torino 155, 30172 Venice, Italy; 8 Department of Microbiology, School of Medicine, Kansai Medical University, 2-5-1 Shinmachi, Hirakata 573-1010, Japan; okumak@hirakata.kmu.ac.jp; 9 Genome Analysis, Institute of Biomedical Science, Kansai Medical University, 2-3-1 Shinmachi, Hirakata 573-1191, Japan; higasa@genome.med.kyoto-u.ac.jp (K.H.); yasukocy@hirakata.kmu.ac.jp (Y.Y.)

**Keywords:** herpes simplex virus type I, Epstein–Barr virus, Raman spectroscopy, glycoproteins, surface pH, secondary protein structure

## Abstract

Raman spectroscopy was applied to study the structural differences between herpes simplex virus Type I (HSV-1) and Epstein–Barr virus (EBV). Raman spectra were first collected with statistical validity on clusters of the respective virions and analyzed according to principal component analysis (PCA). Then, average spectra were computed and a machine-learning approach applied to deconvolute them into sub-band components in order to perform comparative analyses. The Raman results revealed marked structural differences between the two viral strains, which could mainly be traced back to the massive presence of carbohydrates in the glycoproteins of EBV virions. Clear differences could also be recorded for selected tyrosine and tryptophan Raman bands sensitive to pH at the virion/environment interface. According to the observed spectral differences, Raman signatures of known biomolecules were interpreted to link structural differences with the viral functions of the two strains. The present study confirms the unique ability of Raman spectroscopy for answering structural questions at the molecular level in virology and, despite the structural complexity of viral structures, its capacity to readily and reliably differentiate between different virus types and strains.

## 1. Introduction

Herpes simplex virus Type 1 (HSV-1) and Epstein–Barr virus (EBV) belong to a series of nine known human herpesvirus types in the herpes family and are among the most common viruses affecting humans [1,2,3]. HSV-1 is predominantly associated with oral and facial infections, such as cold sores and herpes labialis, but could also cause genital herpes and other infections [4]. On the other hand, EBV is best known as the cause of infectious mononucleosis and is also associated with lymphoproliferative diseases and higher risks for autoimmune diseases, various cancers (including Burkitt lymphoma, Hodgkin’s lymphoma, and nasopharyngeal carcinoma), and multiple sclerosis [4,5].

Despite sharing a number of structural similarities, HSV-1 and EBV viruses also present profound differences. HSV-1 and EBV differ in terms of their genetic material, the former presenting a linear, double-stranded DNA genome, while the latter includes a larger genome, with a circular, double-stranded DNA. In both HSV-1 and EBV, a protein icosahedral capsid and a tegument surround the DNA genome, the overall structure being in turn enclosed within a lipid envelope. However, the presence of viral glycoproteins intertwined with the lipid envelope in EBV, which are essential to infection of the host cell, represent a main structural difference. Their presence enhances binding, viral entry, and immune evasion functionalities. [6] Structural differences at the molecular scale are also linked to the different mechanisms that HSV-1 and EBV viruses adopt in viral pathogenesis, latency, and reactivation [7].

Unfolding the structures of viruses at the molecular level represents the first step in decrypting their common ancestry, understanding their modus operandi, and, accordingly, conceiving structure-guided antiviral drug design [8,9]. The recent flourishing of key analytical techniques in structural biology capable of visualizing and analyzing intact viruses, such as X-ray analyses at near atomic resolution and cryoelectron microscopy [10,11], has added immense value to virology research. However, beyond structural visualization, an improved understanding is needed for unfolding molecular details in complex viruses, which calls for specific analytical procedures effective in “anatomizing” the virus structure into well-defined subunits or substructures [12]. In this context, X-ray analyses can fully resolve only coat protein subunit structures, while much of the virion nucleic acid usually remains too disordered to provide useful diffraction data [13]. 

Raman spectroscopy represents a unique analytical tool in structural virology, being long known for its ability to unravel the makeup of protein, carbohydrate, and nucleic acid constituents of intact viruses upon exploiting the vibrational light scattered from their respective molecules [14,15,16,17]. In our previous studies of viral strains by Raman spectroscopy [18,19,20,21,22,23], we have exploited the Raman method for a fast identification of virus variants/sub-variants and proposed a barcode specially tailored on the Raman spectrum in order to facilitate electronic recordkeeping and to translate molecular characteristics into information rapidly accessible by users. The Raman analyses allowed us to locate structurally sensitive spectral domains in correspondence of signals belonging to sulfur-containing amino acid rotamers, hydrophobic interactions of tyrosine and tryptophan phenol rings, apparent fractions of purine and pyrimidine bases, and protein secondary structures.

In this study, we build upon those previous studies and analyze with the same analytical approach the molecular structures of HSV-1 and EBV. The present Raman data build upon the present knowledge of herpesviruses and their vibrational characterizations [24,25,26,27] while providing, as an original contribution, an almost instantaneous “multi-omic snapshot” of the viruses’ structures. Through Raman analyses, we attempt to newly and promptly unfold structural aspects, such as differences in viruses’ surface pH and glycoprotein amount/structures, which are difficult to determine and hardly found in the available literature. In addition to the above-mentioned structural domains, we shall illustrate here the great potential of Raman spectroscopy in investigating the structure and organization of glycoproteins and of the complex matrices they can form. More specifically, the present Raman analysis gives fresh insights into the complex behavior of these large molecules, thus shedding light on how they collectively contribute to the viral entry process, attachment to host cells, and immune evasion strategies employed by different viruses. Given the crucial role played by glycoproteins in the initial stages of EBV infection, especially in mediating viral attachment to host cells, understanding their spectroscopic features might clarify yet unknown aspects of their structure and function. This could in turn pave the way to future developments in therapeutic interventions aimed at inhibiting their functionality. From a more general viewpoint, the present Raman analyses could contribute to improving understanding of the molecular structure and functions of herpesviruses and giving researchers new hints for developing targeted antiviral strategies and vaccines against EBV.

## 2. Results

### 2.1. Average Raman Spectra and Assignments of Deconvoluted Sub-Bands

**Figure 1:**
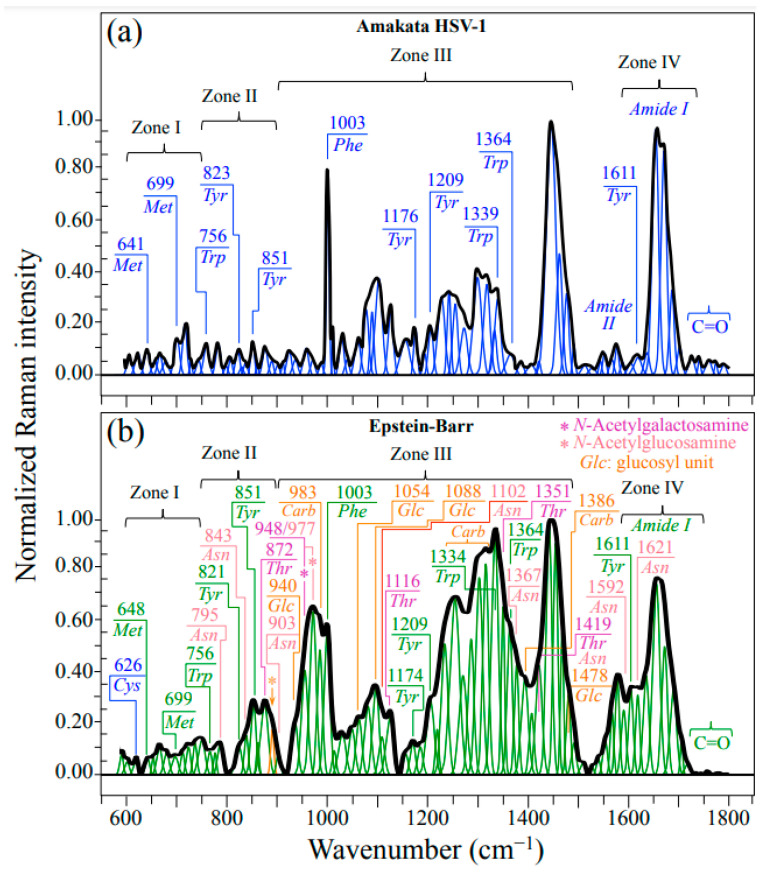
Raman spectra average of 10 collected at distinct locations on each HSV-1 (**a**) and EBV (**b**) sample. The spectra are deconvoluted into Lorentzian–Gaussian sub-band components. Abbreviations are defined in text. Labels in inset indicate sub-band positions in units of cm^−1^. A fingerprint band for β(1–6)-linkages was located at 893 cm^−1^ (C–O–C stretching) and labeled with an arrow/orange asterisk (cf. forthcoming Section 3.2).

Figure 1a,b show normalized Raman spectra (average of 10 collected at distinct locations) for HSV-1 and EBV samples, respectively. As seen at a glance, the two spectra appear extremely different to each other with respect to their overall morphology, the EBV spectrum being generally more intense and populated, especially in correspondence of the intermediate and high wavenumbers. In those spectral areas, the presence of glycoproteins is indeed expected to give a strong signal contribution because of the presence in their structure of carbohydrate molecules (cf. bands labeled as *Carb* in Figure 1b). Strong *Carb* signals could be found at ~983 cm^−1^ (C–O stretching in ring structure) and in the interval 1250~1360 cm^−1^ (CH_2_ twisting and wagging), in addition to a relatively strong signal at 1386 cm^−1^, which was conspicuously missing in the spectrum of HSV-1 (cf. Figure 1a,b) [28]. It should be noted that the spectrum of HSV-1 presented a prominent group of signals in the interval 1400~1500 cm^−1^ (cf. Figure 1a); those signals find a clear correspondence in the EBV spectrum (cf. Figure 1b) and were assigned to CH_2_ bending in proteins [29]. Despite the similarity in CH_2_ protein bands between HSV-1 and EBV, the strong signals related to carbohydrate bands from glycoproteins in EBV are perhaps the most striking evidence for the quite different structures of these two viruses yet belonging to the same family. In addition to the above-mentioned band at 983 cm^−1^, also related to the carbohydrate structure in glycoproteins are additional signals peculiar to the glucosyl (*Glc*) units in the EBV spectrum. Additional *Glc* bands in the EBV spectrum could be located at 940, 1054, and 1088 cm^−1^ (C–C and C–O stretching in ring structure), and 1478 cm^−1^ (OH in-plane bending) [28].

Despite strong signal overlapping, bands belonging to threonine (*Thr*) and asparagine (*Asn*) could be seen particularly prominently in the EBV spectrum. A machine learning analysis (cf. Section 4.2 and [22,30,31]) located bands mainly (i.e., >85%) contributed by *Thr* at 872 cm^−1^ (C–C–N stretching), 1116 cm^−1^ (NH_3_ rocking), 1351 cm^−1^ (CH deformation), and 1419 cm^−1^ (COO^–^ symmetric stretching) (cf. labels in inset to Figure 1b) [32]. On the other hand, a similar procedure located signals mainly contributed by *Asn* at 795 cm^−1^ (CH_2_ rocking), 843 cm^−1^ (NH_2_ out-of-plane bending), 903 cm^−1^ (C–C stretching), 1102 cm^−1^ (NH_2_ rocking), 1367 cm^−1^ (CH symmetric bending), 1419 cm^−1^ (CH_2_ scissoring; shared with Thr), 1592 cm^−1^ (NH_2_ stretching), and 1621 cm^−1^ (NH_2_ bending) (cf. labels in inset to Figure 1b) [33]. The above bands are either absent or significantly weaker at the corresponding locations of the HSV-1 spectrum (cf. Figure 1a). The rationale for the strong presence of *Thr* and *Asn* bands will be discussed in the forthcoming Section 3.2.

**Figure 2:**
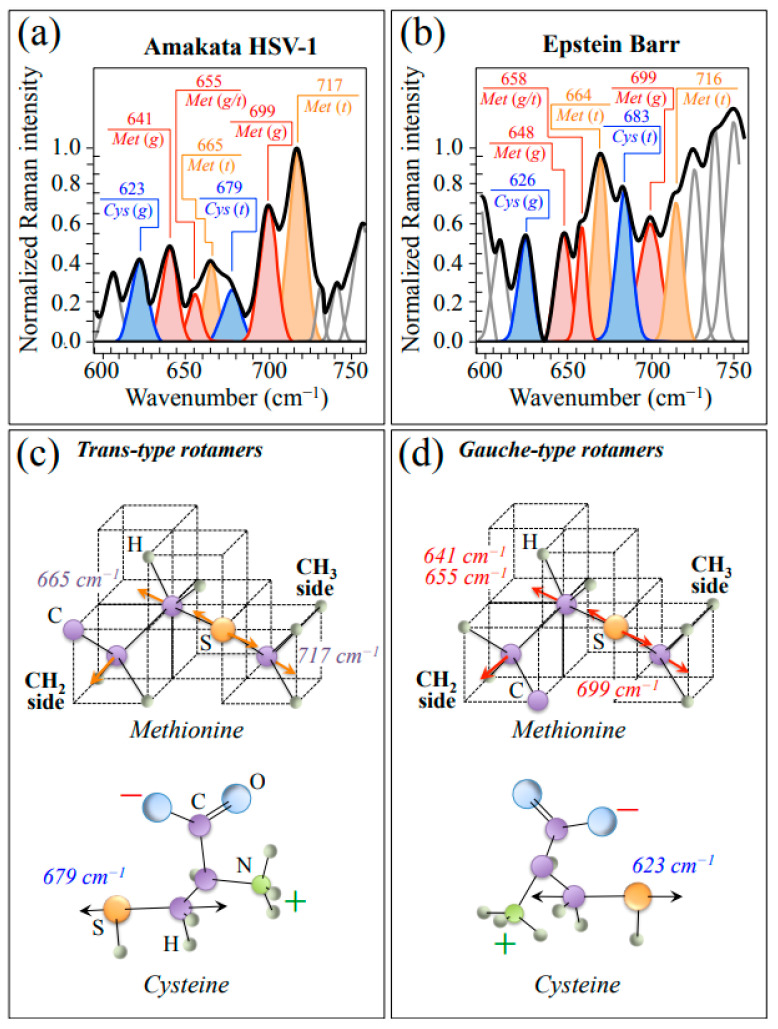
Average spectra in Zone I (600~750 cm^−1^) as recorded with high spectral resolution for HSV-1 (**a**) and EBV (**b**) samples. This zone is dominated by stretching of C–S bonds in different rotamers of S-containing amino acid residues (cf. abbreviations in text). Labels in inset give wavenumbers at maximum of sub-bands in units of cm^−1^. In (**c**,**d**), schematic drafts are given with the prominent vibrational modes methionine and cysteine molecules with *trans* and *gauche* symmetry, respectively.

In order to proceed into more detailed analyses, HSV-1 and EBV spectra were collected with high spectral resolution in restricted spectral zones. Enlarged average spectra in Zone I, representing the wavenumber interval 600~750 cm^−1^, are shown in Figure 2a,b for HSV-1 and EBV, respectively. This spectral zone is dominated by signals related to stretching of C–S bonds in different rotamers of S-containing amino acid residues, namely, methionine (*Met*) and cysteine (*Cys*). Molecules of these amino acid residues with different symmetries, referred to as trans (*t*) and gauche (*g*), are schematically drawn in Figure 2c and Figure 2d, respectively, together with the respective wavenumbers at the maximum of their C–S bands. As can be seen from comparing the spectra in Figure 2a,b, both HSV-1 and EBV basically present the same *Met* and *Cys* sub-bands, although with clearly different relative intensities. Such diversity relates to differences in both rotameric volume fractions and sequences in the respective protein structures and appears to be a prominent factor in characterizing the diversity of the two viral structures.

**Figure 3:**
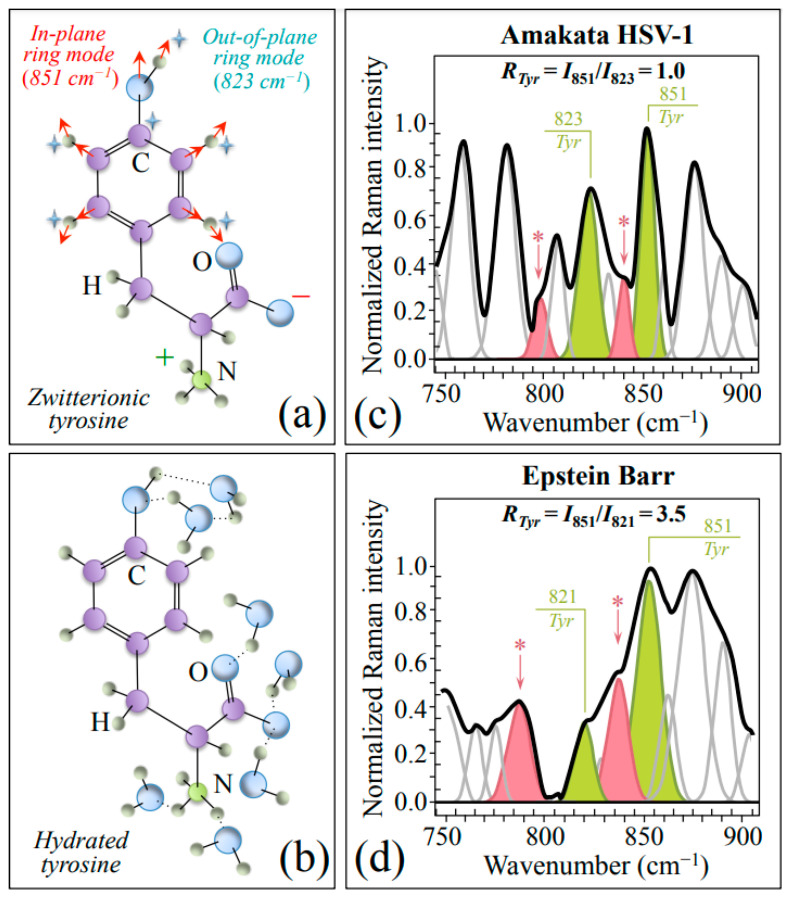
Schematic drafts and prominent vibrational modes of zwitterionic (**a**) and hydrated (**b**) tyrosine. In (**c**,**d**), average spectra in Zone II (750~900 cm^−1^) are given as recorded with high spectral resolution for HSV-1 (**a**) and EBV (**b**) samples. This zone contains signals from two specific ring vibrations of tyrosine residues, as shown in (**a**), from which the tyrosine ratio, *R_Tyr_* = *I*_851_/*I*_823_, was computed (cf. values in inset). Asparagine molecules mainly contribute bands at 795 and 843 cm^−1^, labeled with asterisks.

Zone II (750~900 cm^−1^) contains a strong doublet from the phenyl ring of tyrosine (*Tyr*) aromatic residues (in-plane and out-of-plane ring vibrations at around 851 and 823 cm^−1^, respectively) [34]. As previously discussed by various authors [34,35], the relative intensity of the doublet strongly depends on the environmental pH conditions, namely, whether *Tyr* residues are in zwitterionic or hydrated states (cf. schematic drafts in Figure 3a and Figure 3b, respectively). Figure 3c,d show Zone II as collected with high spectral resolution for HSV-1 and EBV samples, respectively. The low-wavenumber band of the doublet is found in slightly different positions in different virus samples, while the so-called *Tyr* ratio, *R_Tyr_ = I*_851_*/I*_823_, is 3.5 times higher in EBV as compared to HSV-1 (3.5 vs. 1.0; cf. labels in inset to Figure 3c,d). This bold difference, which is representative of a perturbation of the benzene ring symmetry, arises from a strong decrease in relative intensity of the low-frequency band as a fingerprint for a more acidic pH at the interface between EBV virions and the surrounding environment [34,35]. As already mentioned, spectral Zone II is also comprehensive of two prominent *Asn* bands at 795 and 843 cm^−1^ (cf. bands labeled with asterisks in Figure 3c,d). These bands, which are stronger in the EBV spectrum, preserve their relative intensity as an indirect proof of being prominently contributed by *Asn* in both viruses.

**Figure 4:**
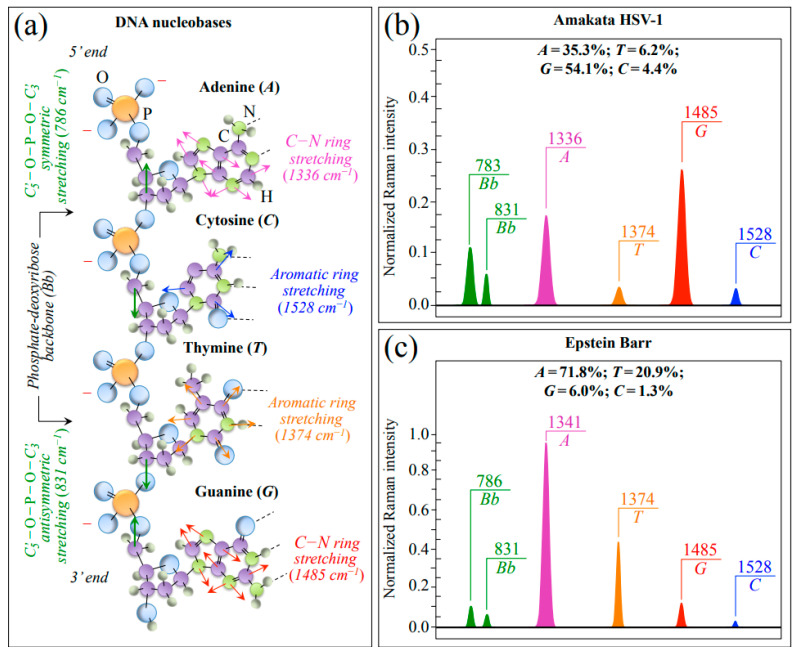
(**a**) Schematic draft of the linked structure of DNA nucleobases with the spectroscopic fingerprints from purine and pyrimidine rings (cf. labels in inset); in (**b**,**c**), Raman sub-bands from purine and pyrimidine nucleobases are extracted from Zone III of the average spectra of HSV-1 and EBV, respectively. Nucleobase relative fractions computed from sub-band areas are given in inset. Signals representing stretching modes of phosphodiester backbone bonds (cf. abbreviations given in text) were also extracted from HSV-1 and EBV spectra; a comparison among these latter bands showed similar intensity ratios in different viral samples (cf. *Bb* doublets drawn in (**b**,**c**)).

In addition to the most intense Raman signal from phenylalanine (*Phe*) at ~1003 cm^−1^ (symmetric ring breathing), Zone III also includes ring-related signals mainly assignable to DNA purine and pyrimidines. Bands representing stretching of heterocyclic aromatic rings that belong to pyrimidines can be found at 1528 and 1374 cm^−1^ for cytosine (*C*) and thymine (*T*), respectively [36,37,38,39]. Conversely, cumulative C–N stretching signals in imidazole and pyridine rings of purines can be found at 1336 and 1485 cm^−1^ for adenine (*A*) and guanine (*G*), respectively. [40] Two additional signals related to the DNA structure are found at ~786 and ~831 cm^−1^ (i.e., in Zone II; cf. Figure 3c,d). These latter signals arise from symmetric and antisymmetric stretching modes of C–O–P–O–C phosphodiester bonds in the phosphate-deoxyribose backbone (*Bb*), respectively [41]. Figure 4a shows a schematic draft of the linked structure of DNA nucleobases with the above-mentioned Raman spectroscopic fingerprints from purine and pyrimidine rings. In Figure 4b,c, Raman sub-bands from purine and pyrimidine nucleobases are extracted from average spectra in Figure 1 of HSV-1 and EBV, respectively. Nucleobase relative fractions computed from sub-band areas are given in inset to each figure. The comparison shows clear differences between the two virus samples, as expected from their different genomic structures (cf. labels in inset to Figure 4b,c). Despite some overlap in the EBV spectrum for the signal at 831 cm^−1^ with the NH_2_ out-of-plane bending band of *Asn*, signals representing the stretching modes of phosphodiester (backbone) bonds roughly maintain their relative intensity in both virus spectra as a sign of consistency in the Raman analysis (cf. Figure 4b,c) [28].

**Figure 5:**
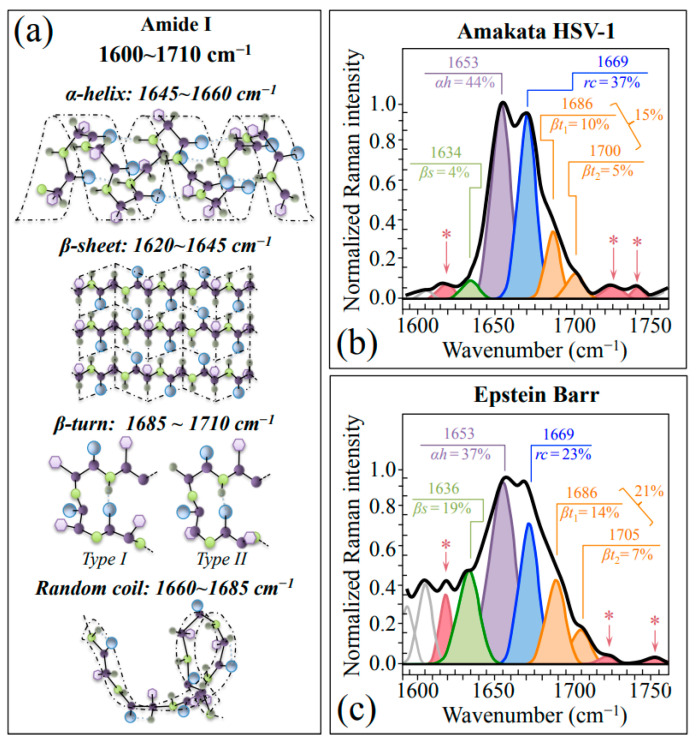
(**a**) Schematic drafts of protein secondary structures with the respective wavenumbers of their Amide I vibration (Zone IV at 1600~1650 cm^−1^). Average Amide I spectra recorded on HSV-1 and EBV are given in (**b**,**c**), respectively. The apparent fractions of each secondary structure are given in inset together with the respective wavenumbers in units of cm^−1^. Additional signals at ~1620, 1722, and >1740 cm^−1^ (cf. deconvoluted bands marked with asterisks in (**b**,**c**)) are related to asparagine and C=O stretching in amino acid molecules.

Finally, Zone IV (1600~1750 cm^−1^) was analyzed. This spectral zone includes the Amide I region, which is representative of the secondary structures of viral proteins. Following spectral deconvolution, Amide I band components from proteins in both HSV-1 and EBV samples can be assigned to β-sheet (*βs*; at 1634~1635 cm^−1^), α-helix (*αh*; at ~1653 cm^−1^), random coil (*rc*; at ~1699 cm^−1^), and Type I and Type II β-turn rotamers (*βt_1_* and *βt_2_*; at ~1686 and 1700~1705 cm^−1^, respectively) (cf. drafts of secondary structures in Figure 5a) [42]. A comparison between the Amide I spectra of HSV-1 (Figure 5b) and EBV (Figure 5c) reveals several important differences, as follows: (i) apparently stronger relative intensities of β-sheet (cf. relative fractions in inset) could be recorded in EBV as compared to HSV-1; however, the ~1636 cm^−1^ band of EBV could also be partly contributed by C=O stretching/NH_3_ antisymmetric bending in *Asn* [33]; (ii) fractional reshuffling apparently occurred mainly at the expenses of random coil, whose fraction in EBV is about 1/3 lower than that found in HSV-1; (iii) fractional increases were found in EBV for both β-turn sub-bands, although also those bands might include contributions by C=O stretching vibrations in *Asn* (cf. later discussion) [33]; (iv) if β-turn rotameric fractions in EBV could actually be considered as the main contributors of bands at ~1686 and ~1700 cm^−1^, a similar trend in rotameric fractions could be recorded in both HSV-1 and EBV samples; and, (v) three additional signals were found in the Amide I region: at ~1620, 1722, and >1740 cm^−1^ (cf. deconvoluted bands marked with asterisks in Figure 5b,c); the former signal being already assigned to *Asn* (cf. above), while the latter two being generically related to C=O stretching but hardly assignable to specific molecules.

For readers’ convenience and in an attempt to give an overview of the complex outputs of Raman analyses, we compiled Table 1, which summarizes the principal spectroscopic differences between the two studied herpesviruses. In this table, the main characteristics noticed in Figure 1, Figure 2, Figure 3, Figure 4 and Figure 5 are included and compared.

### 2.2. PCA Statistical Analysis and Raman Barcoding

**Figure 6:**
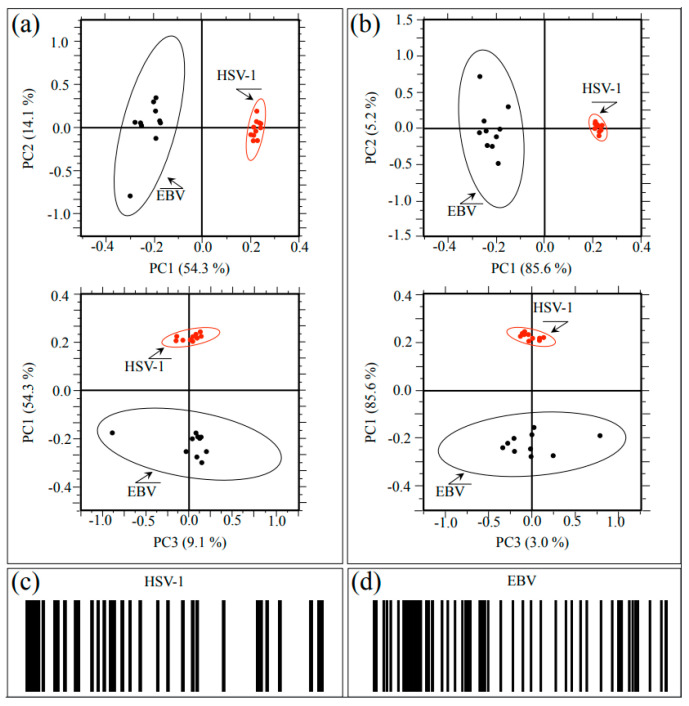
(**a**) Correlation matrix plots of PC2 vs. PC1 and PC1 vs. PC3 and (**b**) covariance matrix plots of the same principal components for both investigated herpesviruses. PC1 is mainly influenced by the relative intensity of the spectrum with respect to the point of normalization, while PC2 and PC3 directly relate to both glycomics and proteomics of the virions; (**c**,**d**) give the Raman barcodes computed from sub-band sequences collected on HSV-1 and EBV samples, respectively.

Raman spectra were collected at ten different locations (10 μm^2^ in size) selected on each HSV-1 and EBV sample. The obtained 20 spectra were then treated with PCA analyses. Figure 6 shows plots of the second vs. first and first vs. third principal components (i.e., PC2 vs. PC1 and PC1 vs. PC3; correlation matrix and covariance matrix in Figure 6a and Figure 6b, respectively) for both studied herpesviruses. PC1 is mainly influenced by the relative intensity of the spectrum with respect to the point of normalization, namely, the most intense band of the recorded spectrum, which differs for the two viruses (cf. Figure 1a,b). Even if accounting for more than 50% of the variance among the spectra, the PC1 component gives little information on the conformation of proteins (including glycoproteins), amino acid residues, and genetic material. On the other hand, PC2 and PC3 directly relate to both glycomics and proteomics of the virions.

The plots show that PCA analyses succeeded in distinguishing between HSV-1 and EBV spectra, although the 10 representative spectra of the EBV showed a significantly higher scatter as compared to the HSV-1 ones. At this stage of the Raman investigation, it is not clear the reason for the high scatter noticed for the EBV spectrum. However, it is believed that the glycomic and proteomic variations noticed in this virus could actually arise from intrinsic compositional fluctuations of the glycoprotein structures in the sample. In summary, the application of PCA statistical approach, which reduces the dimensionality of the Raman data matrix to two orthogonal variables, successfully located the investigated herpesviruses.

We also applied a Raman barcoding approach for virus identification, following a methodology proposed in our previous studies [20,22,30,31,43,44] (regarding the proposed method of constructing a barcode from a deconvoluted Raman spectrum, cf. Section 4.3). The Raman barcode, which represents an alternative approach to chemometric PCA analyses, enables capturing structural details with deeper sensitivity as compared to PCA analyses and, in some cases, allows overcoming speciation deficiencies. The Raman barcodes computed from sub-band sequences collected on HSV-1 and EBV spectra are given in Figure 6c and Figure 6d, respectively. Similar to PCA analysis, barcodes clearly reflected the differences between the Raman spectra of HSV-1 and EBV. As an advantage of the barcode approach as compared to PCA analyses, one could consider that the latter deals with the mere spectral morphology, while the former retains molecular scale information. Spectral differences arising from the different metabolite structures of the two viruses, once translated into line patterns, make multi-omic characteristics easily readable by electronic devices through using appropriate apps.

## 3. Discussion

### 3.1. Raman Fingerprints of Structural Differences between HSV-1 and EBV

As briefly anticipated in the introduction, HSV-1 and EBV are distinct viruses belonging to the herpesvirus family; they share some similarities but also notable differences in their structures. Both viruses are enveloped, meaning they have an outer lipid bilayer membrane surrounding the capsid, but only the EBV virus contains a copious amount of viral glycoproteins. As shown in Figure 1b, this characteristic leaves unambiguous fingerprints of carbohydrate structures in the EBV Raman spectrum. The preponderant glycoprotein of EBV, which is referred to as gp350/220, plays a fundamental role in both functions of binding and entering the target cell [45]. Additional glycoproteins, referred to as gB, gH, and gL, are essential for the fusion of the viral envelope with the host cell membrane during viral entry, while the BMRF-2 protein plays a role in immune evasion by inhibiting the complement system, which is part of the host’s innate immune response [46]. Both the above-mentioned sub-units gp350 and gp220 include sugar molecules in their structure, which are derived from the same gene but undergo differential post-translational modification. The former subunit, which is the larger of the two, forms a homodimer, meaning two gp350 molecules associate with each other to create a stable complex. On the other hand, the gp220 subunit forms a disulfide-linked heterodimer with gp350, which stabilizes the overall structure of the gp350/220 protein complex [47]. Although the exact structure of gp350/220 has not yet been fully resolved at the atomic level, studies using cryo-electron microscopy and other techniques have provided insights into its overall architecture [48,49]. Those studies showed multiple domains, including a C-terminal membrane-spanning domain, an N-terminal globular domain, and several repeating regions. The N-terminal domain of gp350 is the main responsible for receptor binding, while the repeat regions contribute to the stability and antigenic properties of the protein. The membrane-spanning domain anchors gp350/220 in the viral envelope, allowing it to protrude from the viral surface. The present Raman experiments recorded a higher fraction of β-sheet secondary structure in EBV as compared to HSV-1. The low pH at the EBV surface, as revealed by its higher Raman *Tyr* ratio (cf. Figure 3), induces a high level of amino acid protonation, a chemical circumstance that was reported by other authors to stabilize extended β-sheet conformations [50]. Extended β-sheet conformations are, for example, found in the inherently fusogenic glycoprotein B (gB) of the EBV envelope (i.e., domain IV of gB is made entirely of β-sheets) [51]. The amount of gB in the EBV envelope is tightly linked to the ability of the virus to infect cells, and the EBV strains that have more envelope-bound gB exhibit increased infectivity even against cells normally refractory to EBV infections [52,53].

**Figure 7:**
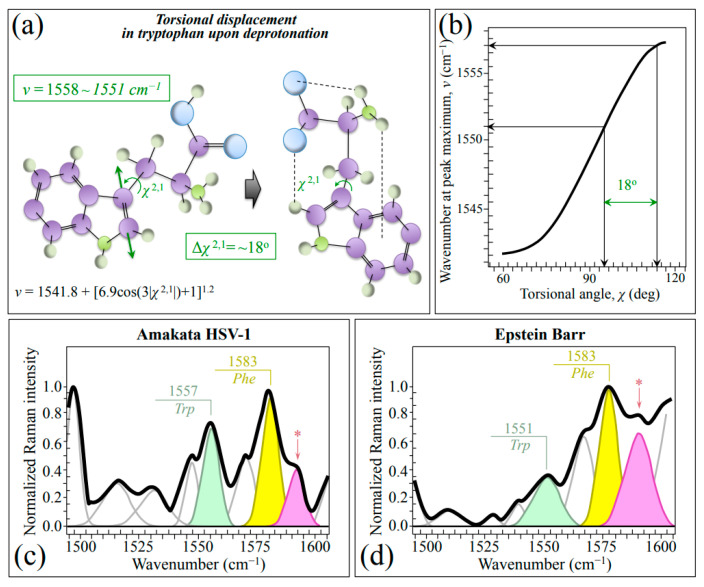
(**a**) Schematic draft of torsional displacements in the tryptophan molecule as a consequence of deprotonation and (**b**) plot of torsional angle, *χ*, as a function of wavenumber position, *ν*, of the C=C stretching signal in *Trp* pyrrole ring (cf. equation in inset to (**a**) and computed torsional displacement, Δ*χ*^2,1^ value in (**a**,**b**); both equation and plot are redrawn from [54]). In (**c**,**d**), average spectra in the wavenumber region 1500~1600 cm^−1^ for HSV-1 and EBV are shown, respectively (cf. abbreviations given in text); the band labeled with an asterisk belongs to asparagine (NH_2_ stretching).

The spectral region in the wavenumber interval 1510~1570 cm^−1^ (cf. labels in Figure 1a,b), which is referred to as the Amide II region, arises from the out-of-phase combination of in-plane N–H deformations with C–N stretches. However, these vibrations can only generate quite weak signals in non-resonant Raman measurements [54], which is indeed the case here. Nevertheless, we observed clearly different signals in this region for Raman spectra from the two investigated herpesviruses. Since they overlap with neither carbohydrate nor lipid bands [28,55], these signals are precious indicators of the difference in their surrounding environment and N-terminal domains. A weak but yet well detectable band in this spectral zone is found at 1542~1557 cm^−1^. This band, which could mainly be assigned to the *Trp* indole ring, represent a hybrid vibrational mode mainly contributed by C=C stretching in the pyrrole ring but also including minor contributions from N–C stretching and C–H bending (cf. schematic draft of *Trp* molecule in Figure 7a) [54,56]. Its changes in wavenumber, ν, are quantitatively related to variations of the torsional angle, χ^2,1^, that defines the orientation of the indole ring with respect to the amino acid backbone (Figure 7b) [54]. We found that the wavenumber at maximum, ν, of this *Trp* band significantly shifted toward lower wavenumbers in EBV as compared to HSV-1 (from 1557 to 1551 cm^−1^; cf. Figure 7c,d). According to the plot in Figure 7b [54], a torsional displacement Δ*χ*^2,1^=18° could be computed. The observed spectral shift, and the structural torsion it represents, reflects a more acidic environment, thus confirming the full protonation and higher hydrophilicity recorded by analyzing the *Tyr* doublet (cf. Figure 3c,d). In other words, the C=C stretching mode of the tryptophan pyrrole ring senses hydrophilic vs. hydrophobic surface characteristics and can be considered, together with the *Tyr* doublet, as a Raman structural marker for the surface chemistry of different herpesviruses. Note that the above observations suggest that EBV virions might need moisture to survive more than the HSV-1 ones, but also that they can more easily be absorbed and penetrate host cells [57]. Moreover, a higher hydrophilicity, and thus a higher capacity to retain water in its surroundings, might help EBV to retain ideal temperature and pH during long periods of latency (cf. also Table 1). These observations link to the different characteristics of HSV-1 and EBV, the former tending to establish latency in sensory neurons, typically in the trigeminal ganglia, while the latter primarily in B-lymphocytes [58]. High hydrophilicity might help EBV to maintain the functions of its latent proteins and to engage different latency programs in different tumors [59], thus allowing the virus to successfully persist in the cytoplasm of infected cells. This point will be further discussed in the forthcoming Section 3.3.

In addition to the already mentioned *Asn* band at 1592 cm^−1^ (NH_2_ stretching; cf. Section 2.1) labeled with an asterisk, an additionally prominent signal in the wavenumber interval 1510~1570 cm^−1^ could be found at ~1583 cm^−1^ (cf. labels in inset to Figure 7c,d). This signal, whose origin resides in the out-of-phase C–C stretching vibrations in the phenyl ring in *Phe*, pairs the in-phase C–C stretching vibrations at ~1606 cm^−1^ [60]. Additional bands at 1530 and 1568 cm^−1^ can be assigned to deoxyguanosine and deoxycytidine triphosphates [61].

Finally, the Raman spectrum also detected clear differences between HSV-1 and EBV in terms of genetic material; HSV-1 showed relatively weaker fingerprint bands for DNA nucleobases as compared to EBV (cf. Figure 4b,c), and this spectral difference is justified by the smaller, linear, and double-stranded DNA genome of HSV-1 as compared to the larger, circular, and double-stranded DNA of EBV. It is not clear at this time whether, and eventually how, the stronger Raman signal of *T* in EBV (as compared to HSV-1), which is not expected by conventional genome analysis, could be related to differences in amount, DNA base-pairing, steric constraints, epigenetic interactions between DNA and small proteins, and/or location of the encoded thymidine kinase enzyme [62]. Whatever the origin of this spectral difference, the strength of the 1485 cm^−1^ *G* band appears to be a powerful spectroscopic marker for distinguishing between EBV and HSV-1 at a glance.

### 3.2. Raman Evidences for O-Glycosylation in EBV

O-glycosylation plays a significant role in EBV infection and immune evasion; the term refers to the attachment of sugar molecules (oligosaccharides) to specific *Thr* and *Asn* residues in proteins. In the context of EBV structural assembly, O-glycosylation is primarily associated with viral glycoproteins, particularly the glycoprotein gp350/220 [63]. As already mentioned above, this glycoprotein is found on the surface of the virus and plays a crucial role in viral attachment and entry into host cells. The O-glycosylation of gp350/220 serves several important functions: (i) it helps the EBV evade the host immune system by shielding viral epitopes from recognition by antibodies; the attached sugar molecules creating a steric hindrance and preventing efficient antibody binding and neutralization of the virus; (ii) it influences its interaction with host cell receptors; by modifying the glycan structures attached to the glycoprotein, the virus can modulate its binding affinity to specific receptors on host cells, thereby affecting viral attachment and entry; and (iii) it is implicated in facilitating the fusion of the viral envelope with host cell membranes. The glycans may directly participate in membrane interactions or contribute to the overall conformation and stability of the glycoprotein, enabling efficient fusion and viral entry into host cells [64].

**Figure 8:**
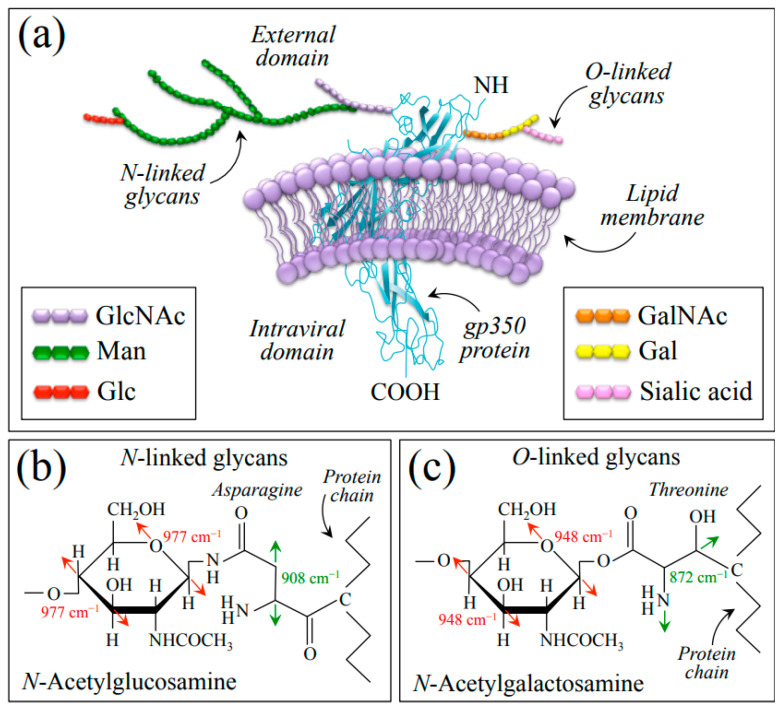
(**a**) Schematic draft of the gp350 protein (drawn according to [66]) embedded in the outer lipid bilayer membrane with *N*-linked GlcNAc (**b**) and *O*-linked GalNAc (**c**) glycan chains. Details of N- and O-linkages, involving asparagine and threonine residues, respectively, are drawn according to [67] (cf. main spectroscopic markers labeled in inset to (**b**,**c**)). List of abbreviations: Gal: Galactose; GalNAc: *N*-Acetylgalactosamine; Glc: Glucose; GlcNAc: *N*-Acetylglucosamine; Man: Mannose.

Understanding O-glycosylation in EBV at the molecular scale is crucial for developing strategies to combat EBV infection and related diseases, and research is ongoing to unravel the specific glycan structures involved, their functional significance, and the interplay between O-glycosylation and other viral and host factors in the context of EBV infection [65]. The present Raman study provides some new information about the vibrational modes of the glycosidic linkages, sugar ring structures, and related modifications present in the EBV glycans. Since the gp350 protein has extensive N- and O-linked oligosaccharide chains, the significant differences observed between the Raman spectra of EBV and HSV-1 are indeed due to the presence of a large number of O-glycosites distributed on the EBV envelope proteins. Figure 8a shows a schematic draft of the gp350 protein (re-drawn according to [66]) embedded in the outer lipid bilayer membrane and its neighborhood with related O-linked chains, which present *N*-acetylglucosamine (GlcNAc) and *N*-acetylgalactosamine (GalNAc) residues at N-linked and O-linked glycan chains, respectively. We have drawn the highly branched N-linked glycans covalently added on *Asn* residues (Figure 8b) and the O-linked glycans added on *Thr* residues (Figure 8c) according to the structure given by Wang et al. [67]. The following main spectroscopic features supported the adoption of this model: (i) the presence of a series of preponderant Raman bands mainly contributed by *Asn* and *Thr*, which were only found in the EBV spectrum (cf. Section 2.1, Figure 1 and Figure 8b,c with fingerprint vibrational modes for *Asn* and *Thr* labeled in inset) [32,33]; and (ii) the presence, only prominent in EBV, of two bands at 948 and 977 cm^−1^ (cf. deconvoluted bands labeled with asterisks in Figure 1b) traceable to C–O/C–C stretching in the glycosyl ring unit of N-GalNAc and N-GlcNAc, respectively (cf. fingerprint vibrational modes in Figure 8b,c as labeled in inset) [28,68].

Serafini-Cessi et al. [69] isolated and analyzed in detail the gp350 EBV envelope glycoprotein and reported about the extensive presence in it of both N- and O-linked oligosaccharide chains. Almost all N-linked oligosaccharides were found to be of a complex type, with a predominance of tri-tetraantennary vs. di-antennary chains. An additional finding was that a significant portion of the tri-tetraantennary chains were bound to an additional branch in β(1–6)-linkage. N-linked oligosaccharides with such a branching pattern were previously associated with neoplastic transformation [70] but never observed in any other herpesvirus glycoprotein. This is indeed one of the most important aspects in EBV research, since it targets the molecular-scale mechanisms by which conversion occurs of a tissue with a normal growth pattern into a malignant tumor.

In this study, we indeed succeeded in detecting a fingerprint band for N-linked oligosaccharide chains in the spectrum of EBV (i.e., *Asn*-linked N-GlcNAc band at 977 cm^−1^; cf. Figure 8b). However, attempts to directly confirm the presence of β(1–6)-linkages by means of Raman spectroscopy resulted to be more problematic. The only possible fingerprint band for this linkage could be located at 893 cm^−1^ (C–O–C stretching) [28] upon machine learning deconvolution (cf. Raman band labeled with an orange asterisk in Figure 1b), but this band component was conspicuously embedded into a crowded spectral zone containing prominent bands from amino acids and thus could not be analyzed quantitatively. Since glycosylation is highly dependent on the host cell, future in situ Raman analyses of herpesvirus-infected living cells could enable an improved understanding of O-glycosylation molecular details, similar to previous studies of Influenza A and HSV-1 virus-inoculated mammalian cells [23,71,72,73]. This study is in line with previously published papers, which showed how Raman spectroscopy could be used in chairside periodontal diagnostics [30,74,75,76], in virus speciation and virion/cell interaction [21,23,73,77,78,79,80], and in early biopsy diagnostics of cancers [81,82,83]. More specifically, the data presented here are preparatory to future Raman assessments, which will allow dissecting specific functionalities of individual viral glycosites and hopefully suggest frameworks for design of glycoprotein vaccines with representative glycosylation.

### 3.3. Comparison and Validation through the Literature Data from Raman and Other Analytical Methods

Tiwari et al. [80] have monitored the replication kinetics of EBV infection in glial cells by means of Raman spectroscopy. Those researchers located unique EBV Raman signals associated with specific biomolecules involved in viral duplication. Enrichment of glycogen polysaccharide, as spotted in cell nuclei upon progressing viral duplication, could be recognized by means of specific Raman fingerprints located in the spectral interval 850~1124 cm^−1^. This feature is indeed exactly matching one of the main spectroscopic characteristics that we report here for the examined EBV strain as a distinct one from the spectral characteristics of the HSV-1 strain.

Another important finding of the present Raman study was the lower pH/higher hydrophilicity of EBV as compared to HSV-1. Two independent Raman fingerprints supported this finding, both reflecting the full protonation state of aromatic amino acids: the value of the *Tyr* ratio, *R_Tyr_ = I*_851_*/I*_823_, 3.5 times higher in EBV as compared to HSV-1 and the significantly lower wavenumber of the C=C stretching band in the *Trp* pyrrole ring for EBV as compared to HSV-1 (1551 vs. 1557 cm^−1^). In a quantitative Raman analysis of *Tyr* doublet in endogenous pentapeptides of the central nervous system as a function of pH, Abdali et al. [84] reported a value *R_Tyr_* = 1.7 at pH 4 and used this result to prove that the *Tyr* residue is exposed to the environment and becomes a donor for H bonds. The *R_Tyr_* value measured in this study for EBV (i.e., *R_Tyr_* = 3.5) thus suggests the *Tyr* residue is embedded in an extremely acidic environment. Protonation of interfacial amino acids in water leads to the formation of pH-dependent electrically charged surfaces in viruses, which govern environmentally assisted virus/cell interactions. [85] In a comprehensive paper describing the process of EBV fusion and penetration into white blood (B) cells, Hutt-Fletcher [86] detailed how virus endocytosis is triggered by the interaction between gp350/220 and CR2 and reported that such interaction requires a low-pH compartment to occur, although low pH itself is not a requirement [87]. Notably, HSV-1 presents no requirement for acidification to trigger fusion to B cells [88]. The present Raman data thus confirm and add new information on the above important notions of different endocytosis processes for different herpesviruses. In particular, our data newly suggest that EBV is capable of intrinsically maintaining a quite low environmental pH (locally) at its surface, which makes its entry into B cells more effective and, more importantly, independent of environmental conditions. Conversely, HSV-1, which does not need acidification for viral entry, experiences an intrinsically higher pH value at its surface, which does not affect endocytosis.

Finally, it should be noted that, while Raman spectra of herpesviruses-infected vs. control cells have already been the object of published work [24,27], Raman spectra of Herpesviruses only (i.e., without cells’ signals) are seldom found and compared in the published literature. Moreover, Raman spectra of viruses are often collected using surface-enhanced Raman spectroscopy (SERS), thus under experimental conditions quite different from those used in the present experiments [89]. In this study, we succeeded in recording the Raman spectrum of HSV-1 and EBV virions without the presence of cells and without using SERS or other signal-enhancing methods, which could potentially alter the virions’ spectrum due to interaction between virions and the metal nanoparticles used for enhancing the signal. The equipment and the procedure by which the present spectra could be recorded are described in Section 4.3.

## 4. Materials and Methods

### 4.1. Virus Samples

Experiments with human herpesvirus 1 (HHV-1; simply referred to as herpes simplex virus Type 1, HSV-1) Amakata strain and Epstein–Barr virus (strain B95-8, human gammaherpesvirus 4 ASM240226v1; simply referred to as EBV throughout the paper) were conducted in a biosafety Level 2 (BSL-2) biocontainment facility according to BSL-2 work practices. The investigated herpesviruses have long been maintained in our laboratory at the Kyoto Prefectural University of Medicine [71,72].

EBV virions were prepared from the B95-8 cell line (monkey lymphocyte cell line producing EBV; kindly provided by Prof. Kazufumi Ikuta, Division of Microbiology, Tohoku Medical and Pharmaceutical University, Sendai, Japan). B95-8 cells were cultured in RPMI1640 (Nacalai Tesque, Inc., Kyoto, Japan) supplemented with 10% heat-inactivated fetal bovine serum and Penicillin-Streptomycin Mixed Solution (Nacalai Tesque, Inc., Kyoto, Japan) at 37 °C, in 5% CO_2_. Briefly, 10 mL of culture supernatant after 5 days of cell confluence was divided into 1 mL in 1.5 mL microtubes and centrifuged at 14,000 rpm for 90 min at 4 °C. After centrifugation, supernatant was removed and the remaining pellet at the bottom was mixed by pipetting with PBS and collected.

### 4.2. Raman Samples and Spectroscopic Procedures

Virions were placed on a glass-bottom dish (MatTek Life Sciences, Ashland, MA, USA) air-dried and fixed 4% paraformaldehyde Phosphate Buffer (Nakalai Tesque, Inc., Kyoto, Japan) at room temperature for 10 min. After virus fixation, samples were washed twice with PBS (–) and then again twice with DW. Virus samples were dried in air and stored at 4 °C until Raman analyses.

Raman experiments were conducted by employing a specially designed spectrometer (LabRAM HR800, Horiba/Jobin-Yvon, Kyoto, Japan) set in confocal mode. A holographic notch filter concurrently provided high-efficiency and high-resolution spectral acquisitions. The wavelength of the incoming laser was 785 nm, with the laser source operating with a laser power of 70 mW. The Raman scattered light was analyzed with a double monochromator connected with an air-cooled charge-coupled device (CCD) detector (Andor DV420-OE322; 1024 × 256 pixels); the grating used in the spectrometer had a resolution of 1800 g/mm. The spectral resolution was ~1 cm^−1^. The acquisition time of a single spectrum was typically 20 s for 3 consecutive acquisitions at the same location to eliminate noise. The laser spot was ~2 μm as focused on the sample through a 50× optical lens. Sets of ten spectra were collected at different locations on each sample (over areas of ~2 mm^2^) and averaged in order to obtain an average spectrum used throughout the analyses.

**Figure 9:**
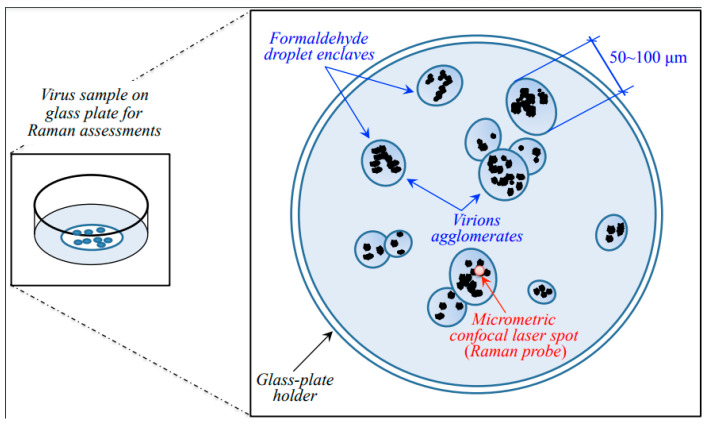
Schematic of the sample setup and sample/probe interaction in Raman spectroscopic assessments.

The spectrum of a sample only consisting of paraformaldehyde was preliminary recorded using exactly the same spectroscopic measurement conditions adopted for virions’ assessments. Under red light, the paraformaldehyde used for fixation only appeared as a broad background, which could easily be eliminated according to a standardized procedure, as explained later in this section. A schematic draft explaining the sample setup and the sample/probe interaction is given in Figure 9.

As seen in the draft of Figure 9, agglomerations of virions were spotted into semi-ellipsoidal pools (enclaves) of paraformaldehyde typically 50~100 μm in diameter. Under the microscope, the agglomerated virions appeared as black/grey “dust” with variable focal positions. Accordingly, the micrometric laser spot was focused at selected locations of strong signal to collect the Raman spectrum. A preliminary *z*-scan was needed to focus the laser exactly on the virions and to maximize their spectrum with respect to the formaldehyde broad background. Spectra were collected on different enclaves and averaged for each sample.

Note that the fortunate circumstance of virions’ agglomeration inside formaldehyde enclaves enabled a significant reduction of signal-to-noise ratio (>10). The noise could further be reduced upon averaging over several successive acquisitions at the same location.

Raman spectra were subjected to baseline subtraction according to the asymmetric least square method. The average spectra were deconvoluted into series of Lorentzian–Gaussian sub-bands using commercial software (LabSpec 4.02, Horiba/Jobin-Yvon, Kyoto, Japan). In performing this deconvolutive procedure, a machine-learning approach was applied, which employed an in-house built automatic solver described in previous studies [22,30,31].

### 4.3. Statistical Analyses and Buildup of Raman Barcodes

Statistical analyses of the Raman data sets were performed according to principal component analysis (PCA) on ten selected spectra per each viral strain acquired at different locations on each sample. PCA analyses were conducted by means of the Origin software platform (OriginPro 2017, OriginLab^®^ Co., Ltd., Northampton, MA, USA) and displayed by means of a set of “summary indices”, referred to as principal components PC1, PC2, and PC3.

**Figure 10:**
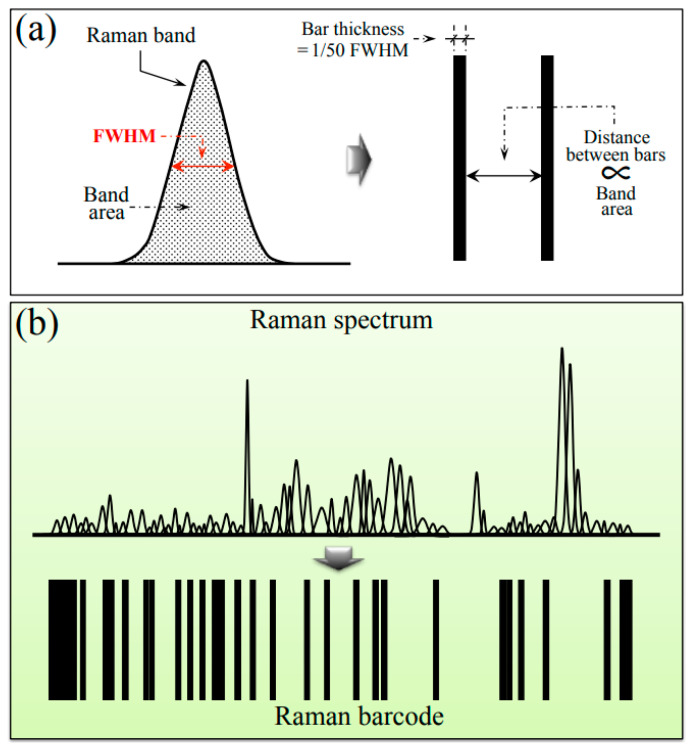
Proposed procedure for converting a deconvoluted Raman spectrum into a barcode: (**a**) conversion of a single sub-band into a bar and (**b**) conversion of the entire spectrum into a barcode.

Barcodes were generated from deconvoluted Raman spectra using the respective sequences of Gaussian–Lorentzian sub-bands. Sub-band sequences were converted into barcodes by means of an algorithm that assigned to each band a line with thickness equal to 1/50 of the sub-band full width at half maximum and a distance from the successive line proportional to the sub-band area. The procedure of barcode construction is briefly explained in the draft of Figure 10a,b, which give the conversion of a single Raman sub-band into a bar and conversion of the entire spectrum into a barcode, respectively). The Raman barcode could enable efficient electronic recordkeeping and increase data accessibility by storing the structural characteristics of different sub-variants to be suitably converted into informative text through apps and user-friendly software.

## 5. Conclusions

The present Raman spectroscopic study of herpesviruses substantiated a number of fundamental characteristics by which HSV-1 and EBV are diversified at the molecular scale. The main spectroscopic differences could be summarized as follows: (i) unlike HSV-1, the Raman spectrum of EBV was dominated by the presence of signal from glycoproteins, which gave strong contributions especially in the middle and high wavenumber regions, due to the presence in their structure of carbohydrate molecules; (ii) two concurrent pH-sensitive spectroscopic features, namely, a significant (i.e., 3.5 times) increase of the *Tyr* ratio, *R_Tyr_ = I*_851_*/I*_823_, and a prominent shift from 1557 to 1551 cm^−1^ of the C=C stretching signal in *Trp* pyrrole ring, converged in proving a significant difference in surface chemistry between the two studied viruses; the EBV being significantly more acidic and hydrophilic at its surface than HSV-1. This intrinsic property ensures the presence of moisture at the virion surface, leading to higher absorption and penetration in host cells, and high survivability during long periods of latency; (iii) the major EBV envelope glycoprotein, gp350, left extensive fingerprints of its *N*- and *O*-linked oligosaccharide chains, the former linked to the protein backbone through an *Asn*/N-GlcNAc unit, while the latter through an *Thr*/N-GalNAc one. Strong signals conducible to *Asn* and *Thr* were only detected in the spectrum of EBV, which also included fingerprint C–O/C–C stretching signals from glycosyl ring units of N-GalNAc and N-GlcNAc molecules (at 948 and 977 cm^−1^, respectively); (iv) as a direct consequence of the above three major structural differences, a PCA analysis enabled us to clearly distinguish EBV and HSV-1 by means of their Raman spectra, thus opening the way to a fast method of Raman diagnostics.

In conclusion, Raman scattering was confirmed to be a powerful tool in viral speciation and metabolite analyses; once coupled with machine learning and statistical analyses, it could become a powerful tool in diagnostics and in locating sites of vulnerability for vaccine development.

## Figures and Tables

**Table 1:** Summary of the main Raman spectroscopic differences found between EBV and HSV-1.

Structural Difference	Raman Fingerprints	Implications
Carbohydrate signals: stronger in EBV than HSV-1	940, 983, 1054, and 1088 cm^−1^ (C−C & C−O stretching in *Glc* ring) 1250~1360 cm^−1^ (CH_2_ twisting & wagging)	These signals reveal the prominent presence of glycoproteins in EBV
Tyrosine ratio, R_Tyr_ = I_851_/I_823_, higher in EBV than HSV-1	851/823 cm^−1^ tyrosine doublet (in-plane & out-of-plane ring vibrations)	Significantly more acidic pH at EBV virion/environment interface
Differences in Raman genomic structures between EBV and HSV-1	1528 (*C*) &1374 (*T*) cm^−1^ (ring stretching in pyrimidines) 1336 (*A*) & 1485 (*G*) cm^−1^ (C−N ring stretching in purines)	Different fractions of purines and pyrimidines as expected from different genome structures
Differences in protein secondary structure: β-sheet/β-turn fractions higher in EBV than HSV-1	Amide I bands: 1634~1636 cm^−1^ → β-sheet 1653 cm^−1^ → α-sheet 1669 cm^−1^ → random coil 1686/1705 cm^−1^ → β-turn	More rigid protein structure in EBV; it also justifies the stronger threonine signals found in EBV
Increased tryptophan torsional angle, χ^2,1^, in EBV as compared to HSV-1	1557 → 1551 cm^−1^ (C=C stretching in tryptophan pyrrole ring)	Higher hydrophilicity in EBV helps to retain temperature and pH during latency

## Data Availability

Data available on request.
